# Quinine exposure and the risk of acute kidney injury: a population-based observational study of older people

**DOI:** 10.1093/ageing/afaa079

**Published:** 2020-05-28

**Authors:** Andrew D S Duncan, Simona Hapca, Nicosha De Souza, Daniel Morales, Samira Bell

**Affiliations:** 1 Renal Unit, Ninewells Hospital and Medical School, Dundee DD1 9SY, UK; 2 Division of Population Health and Genomics, School of Medicine, Ninewells Hospital and Medical School, University of Dundee, Dundee DD1 9SY, UK; 3 Division of Computing Science and Mathematics, Faculty of Natural Sciences, University of Stirling, Stirling FK9 4LA, UK

**Keywords:** *acute kidney injury*, *quinine*, *leg cramps*, *older people*, *epidemiology and pharmacovigilance*

## Abstract

**Objectives:**

to establish and quantify any observable association between the exposure to community prescriptions for quinine and acute kidney injury (AKI) events in a population of older adults.

**Design:**

two observational studies using the same dataset, a retrospective longitudinal cohort study and a self-controlled case series (SCCS).

**Setting:**

NHS health board in Scotland.

**Participants:**

older adults (60+ years) who received quinine prescriptions in Tayside, Scotland, between January 2004 and December 2015. The first study included 12,744 individuals. The SCCS cohort included 5,907 people with quinine exposure and more than or equal to one AKI event.

**Main outcome measured:**

in the first study, multivariable logistic regression was used to calculate odds ratios (ORs) for AKI comparing between episodes with and without recent quinine exposure after adjustment for demographics, comorbidities and concomitant medications. The SCCS study divided follow-up for each individual into periods ‘on’ and ‘off’ quinine, calculating incidence rate ratios (IRRs) for AKI adjusting for age.

**Results:**

during the study period, 273,596 prescriptions for quinine were dispensed in Tayside. A total of 13,616 AKI events occurred during follow-up (crude incidence 12.5 per 100 person-years). In the first study, exposure to quinine before an episode of care was significantly associated with an increased probability of AKI (adjusted OR = 1.27, 95% confidence interval (CI) 1.21–1.33). In the SCCS study, exposure to quinine was associated with an increased relative incidence of AKI compared to unexposed periods (IRR = 1.20, 95% CI 1.15–1.26), with the greatest risk observed within 30 days following quinine initiation (IRR = 1.48, 95% CI 1.35–1.61).

**Conclusion:**

community prescriptions for quinine in an older adult population are associated with an increased risk of AKI.

## Graphical Abstract

**Graphical Abstract:**
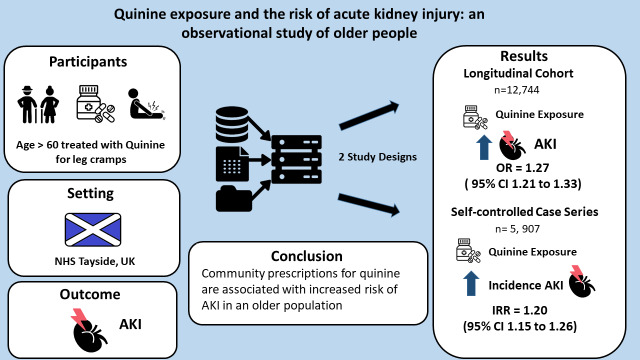

## Key points

- Quinine is commonly prescribed to treat leg cramps
- Acute Kidney Injury (AKI) and leg cramps share a number of risk factors, and quinine itself can rarely precipitate AKI by immune-mediated reactions
- Quinine prescriptions are associated with an increased risk of AKI in older adults

## Introduction

Acute kidney injury (AKI) is associated with a number of negative outcomes including short- and long-term mortality [1–3] and the development of chronic kidney disease (CKD) [3, 4]. In the UK, one in 20 people over 70 years of age experience an AKI each year [5] with the majority of AKI cases developing in the community [6]. Identifying modifiable individual risk factors, such as nephrotoxic medications, is an important target towards which preventative efforts can be directed, particularly in the older population [7]. Cramps are sudden, painful, involuntary contractions of muscles, typically occurring in the calf or foot, which affect half of people over 80 years of age [8, 9]. Although usually idiopathic, certain conditions and medications are associated with leg cramps [8]. A number of these conditions overlap with risk factors for AKI, notably the use of diuretics, CKD, volume depletion and increased age [7, 10]. Quinine is the most commonly prescribed medication in the UK and USA to treat leg cramps [11, 12]. Quinine itself can precipitate AKI by immune-mediated reactions, notably drug-induced thrombotic microangiopathy (TMA) of which it is the most common cause [13, 14]. Quinine-induced TMA is thought to be mediated by antibodies that, in the presence of quinine, target antigens on neutrophils and platelets [15]. Although TMA is an uncommon adverse drug reaction, quinine-induced TMA can be life-threatening with up to 90% of individuals requiring dialysis [16]. Despite leg cramps sharing a number of common risk factors with AKI, and quinine itself known to affect the kidney by rare immune-mediated reactions, the relationship between cramps, quinine and AKI at a population level is unclear. The aim of this study is to establish and quantify any observable association between the exposure to community prescriptions for quinine and AKI events in a population of older adults.

## Methods

We performed two population studies using the same dataset, a retrospective longitudinal cohort study and a self-controlled case series (SCCS), of adults aged 60 years or over who received community prescriptions for quinine in Tayside, a region of Scotland with an approximate population of 400,000. The study period was from 1 January 2004 to 31 December 2015. Further details on methodology are described in Appendix 1.

### First study design

The retrospective longitudinal study cohort included individuals who had received at least one prescription for quinine and had at least two serum creatinine measurements more than 7 days apart after the age of 60 years. A unique follow-up period was calculated for each individual. Follow-up started on the date of the first prescription for quinine or creatinine measurement after the age of 60 years, whichever was earlier, and ended at the end of the study period, death or the date that an individual commenced renal replacement therapy (RRT), whichever occurred first. People receiving RRT at the start of the study period were not included.

### Quinine exposure and episodes of care

The first study design examined the risk of AKI with quinine exposure during ‘episodes of care’. As individuals may have repeated creatinine measurements clustered around a period of illness, creatinine measurements within 7 days of each other were grouped into episodes of care. The 12 weeks prior to each episode of care were examined to identify the presence or absence of community prescriptions for quinine (Appendix 2). The presence of any quinine prescriptions during this time period inferred quinine exposure, either recent or current. The risk of AKI was compared between the episodes of care with and without quinine exposure.

### Statistical analysis

Demographic characteristics and comorbidities are presented as counts and percentages for categorical variables and means and standard deviations or medians and interquartile ranges (IQRs) for quantitative data. A logistic regression model for repeated measures binary data was used to examine the association between quinine prescribing and the risk of AKI. The size of the association was estimated based on odds ratios (ORs), unadjusted and adjusted for demographic characteristics, comorbidities and medication prescribed in the 12 weeks prior to the episode of care. The follow-up time from the start of the study period to the index episode of care and the residual numbers of episodes of care until the index episode (after adjustment for follow-up time) were also included to try and account for the frequency of creatinine measurements that varied between individuals. The model estimates were calculated with clusters defined by patient anonymised identification number to account for correlation in the data. Model variable selection was carried out using the quasi-likelihood information criterion. All analyses were performed using SAS 9.4 [17].

### Second study design

A second study, an SCCS, was also performed to explore further the relationship between the use of quinine and AKI in older people. The SCCS method can be used to study acute recurrent outcomes and transient exposures for which exact timings are available. Individuals act as their own controls with follow-up time divided into periods with and without exposure [18]. The effects of time-invariant confounders, such as sex, location and underlying state of health, are therefore eliminated [18]. The SCCS design has been used previously to examine the relationship between community antibiotic prescribing and AKI [19]. To be included in the SCCS method, individuals must experience the exposure and the outcome of interest; therefore, only individuals from the first study cohort who experienced an AKI during follow-up were included in the SCCS cohort. The inclusion criteria and follow-up were otherwise the same, with observation starting on the date of the first prescription for quinine or creatinine measurement after the age of 60, whichever was earlier, and ending at the end of the study period, death or the date that an individual commenced RRT, whichever occurred first. The observation period for each individual was divided into 5-year age categories (60–64, 65–69, 70–74, 75–79, 80–84, 85–89 and 90+ years). Exposure risk periods (i.e. time periods ‘on’ quinine) started on the date of quinine prescription and ended on the date of prescription + [1.5 × number of tablets dispensed] as 93.7% of prescriptions contained directions that one quinine tablet should be taken once daily. If an individual had successive quinine prescriptions leading to overlapping time periods ‘on’ quinine, these were merged together into a single time period. All other follow-up time in each age category represented baseline time ‘off’ quinine. The exposure risk periods ‘on’ and ‘off’ quinine were further subdivided into initiation and pre-exposure periods, respectively (Appendix 3). The initiation period comprised of the first 30 days after quinine prescription. The pre-exposure period comprised of the 30 days prior to quinine prescription. The date of the first creatinine measurement of an episode of care was used as the date of the AKI event. Incidence rate ratios (IRRs) and 95% confidence intervals (CIs) were calculated comparing the incidence of AKI during the different risk periods, assuming a Poisson distribution and adjusting for age category [20]. All analyses were performed using SAS 9.4 [17].

### SCCS sensitivity analyses

The SCCS method assumes that recurrent outcomes occur independently [18]. AKI increases the risk of an individual experiencing a further AKI. We therefore conducted a sensitivity analysis that examined first AKI events only [18]. People who had experienced an AKI prior to the start of follow-up (according to creatinine measurements from January 2003) were removed, and only the first AKI during follow-up was included in this analysis. Furthermore, when using the SCCS method, the occurrence of an event should not censor the observation period [18]. AKI is associated with mortality and developing the need for RRT. A second sensitivity analysis was performed excluding individuals for whom follow-up ended within 90 days of an AKI event [18].

## Results

Between January 2004 and December 2015, there were 273,596 prescriptions for quinine dispensed to 16,750 people in Tayside. The distribution of quinine prescriptions over the time period is shown in Appendix 4.The median age at first prescription was 69.7 years (IQR 59.5–77.9).

A total of 12,744 people fulfilled the inclusion criteria and were included in the first study cohort. The cohort was predominantly female (62.5% female) with a median age of 70.5 years (IQR 63.9–77.4) at the start of follow-up. Baseline characteristics are outlined in Table 1. A total of 5,037 individuals (39.5%) in the cohort died during the study period and 39 people (0.3%) commenced RRT. People in the cohort had a significant number of comorbidities that increased throughout follow-up with subsequent episodes of care. The prevalence of CKD increased from 10 to 47% by the end of follow-up and the number of people with a history of myocardial infarction, heart failure, cerebrovascular disease or peripheral vascular disease more than tripled.

**Table 1:** Baseline characteristics of the first study cohort

Variable		Frequency (%) *N* = 12,744
Sex	Female	7,968 (62.5)
	Male	4,776 (37.5)
Age	Median (IQR)	70.5 (63.9–77.4)
SIMD	1	2,235 (17.8)
	2	2,108 (16.8)
	3	2,317 (18.5)
	4	3,773 (30.1)
	5	2,118 (16.9)
	Missing data	193
Follow-up	Median in years (IQR)	9.7 (6.2–11.5)
	RRT during study period	39 (0.31)
	Died during study period	5,037 (39.5)
Quinine prescriptions	Total	233,111
	Median per person (IQR)	6 (1–27)
Episodes of care	Total	267,900
	Median per person (IQR)	18 (10–27)
CKD status	eGFR > 60	11,419 (89.6)
	eGFR 30–60	1,201 (9.4)
	eGFR 15–30	117 (0.9)
	eGFR < 15	7 (0.1)
History of previous AKI	Nil	12,120 (95.1)
	Stage 1	470 (3.7)
	Stage 2	111 (0.9)
	Stage 3	43 (0.3)
Comorbidities	Type 1 diabetes mellitus	34 (0.3)
	Type 2 diabetes mellitus	1,574 (12.4)
	Myocardial infarction	606 (4.8)
	Heart failure	417 (3.3)
	Peripheral vascular disease	461 (3.6)
	Cerebrovascular disease	573 (4.5)
	Liver disease	101 (0.8)

Abbreviations: eGFR, estimated glomerular filtration rate (ml/min per 1.73 m^2^); SIMD, Scottish Index of Multiple Deprivation (Quintiles 1-most deprived, 5-least deprived).

IQR presented as Q1–Q3.

Age and comorbidities at start of follow-up.

### Episodes of care and incidence of AKI

A total of 267,900 episodes of care occurred during follow-up. A summary of characteristics by episode of care is presented in Appendix 7. A total of 5,907 people (46.4%) in the study cohort experienced an AKI during follow-up. Of these individuals, the median number of AKI episodes was 2 per person (IQR 1–3). The overall incidence rate of AKI was 12.5 events per 100 person-years.

### Risk of AKI associated with quinine exposure

Crude and adjusted ORs for the association between AKI and each variable modelled in the first study design are presented in Table 2. The crude and adjusted ORs for the risk of AKI occurring during an episode of care with exposure to quinine was OR 1.45 (95% CI 1.39–1.53) and OR 1.27 (95% CI 1.21–1.33), respectively.

**Table 2:** Unadjusted and adjusted OR of AKI by variable

Variable		Unadjusted OR (95% CI)	Adjusted OR (95% CI)
	Quinine exposure	1.45 (1.39–1.53)	1.27 (1.21–1.33)
Demography	Age (per 5-year increase)	1.25 (1.23–1.27)	1.18 (1.16–1.21)
	Sex M vs F	1.01 (0.95–1.07)	1.07 (1.01–1.12)
	SIMD	0.97 (0.95–0.99)	0.97 (0.95–0.99)
Number and time of episode	Time of episode (per 1-year increase)	1.03 (1.02–1.04)	0.99 (0.98–1.00)
	Number of episodes of care	1.01 (1.01–1.01)	0.99 (0.99–1.00)
History of previous AKI	Stage 1	2.88 (2.74–3.02)	2.23 (2.11–2.35)
	Stage 2	4.14 (3.83–4.47)	3.01 (2.77–3.28)
	Stage 3	4.69 (3.98–5.54)	3.69 (3.14–4.34)
CKD status	eGFR 30–60	1.93 (1.83–2.03)	1.16 (1.09–1.22)
	eGFR 15–30	2.88 (2.65–3.13)	1.19 (1.08–1.31)
	eGFR < 15	5.48 (3.51–8.57)	2.73 (1.65–4.54)
Other prescriptions	ACEI	1.04 (0.99–1.10)	0.98 (0.93–1.02)
	ARB	0.92 (0.86–0.99)	0.91 (0.85–0.97)
	Diuretic	1.66 (1.57–1.76)	1.19 (1.12–1.25)
	NSAID	0.95 (0.89–1.02)	1.29 (1.21–1.38)
	Trimethoprim	2.28 (2.14–2.43)	1.87 (1.76–1.99)
	Number of other drugs	1.13 (1.12–1.14)	1.05 (1.04–1.07)
Comorbidities	Type 1 diabetes mellitus	1.38 (0.86–2.21)	1.34 (0.96–1.86)
	Type 2 diabetes mellitus	1.07 (1.00–1.14)	0.87 (0.82–0.92)
	Myocardial infarction	1.83 (1.71–1.96)	1.11 (1.04–1.19)
	Heart failure	2.65 (2.48–2.84)	1.45 (1.36–1.55)
	Peripheral vascular disease	2.19 (2.03–2.35)	1.37 (1.28–1.47)
	Cerebrovascular disease	2.02 (1.89–2.17)	1.35 (1.27–1.44)
	Liver disease	1.89 (1.61–2.23)	1.61 (1.35–1.93)

Abbreviations: ACEI, angiotensin-converting enzyme inhibitor; ARB, angiotensin II receptor blocker; eGFR, estimated glomerular filtration rate (ml/min per 1.73 m^2^); NSAID, non-steroidal anti-inflammatory drug; SIMD, Scottish Index of Multiple Deprivation (1-most deprived, 5-least deprived).

Trimethoprim refers to trimethoprim-containing antibiotics including co-trimoxazole. Age at start of follow-up. Time of episode is from start of study period until episode of care. Number of episodes of care is the number of previous episodes of care until the index episode using residuals after adjustment for time of episode.

### Second study design

The SCCS study included the 5,907 individuals who experienced an AKI in the first study cohort. Baseline characteristics of the SCCS cohort are outlined in Appendices 8 and 9. The cohort was predominantly female (62.6% female) with a median age of 73.6 years (IQR 67.1–79.8) at the start of follow-up. During the observation period, a total of 13,616 AKI events occurred. The baseline incidence rate of AKI ‘off’ quinine was 26.7 events per 100 person-years. Total follow-up time ‘on’ quinine was 14,535 years compared to 33,378 years ‘off’ quinine.

### IRRs

The crude and adjusted IRRs of AKI associated with quinine exposure periods were IRR 1.21 (95% CI 1.15–1.27) and IRR 1.20 (95% CI 1.15–1.26), respectively. The adjusted IRR remained significant following sensitivity analyses restricted to first AKI events only (5,471 individuals, IRR = 1.21, 95% CI 1.15–1.26) and removal of individuals who experienced AKI within 90 days of the end of follow-up (3,541 individuals, IRR = 1.27, 95% CI 1.19–1.34). When the initiation and pre-exposure risk periods were examined, the IRR for AKI was greatest within 30 days following quinine initiation (IRR = 1.48, 95% CI 1.35–1.61, Table 3). There was no difference in the relative incidence of AKI during the pre-exposure period compared to the baseline period (IRR = 0.98, 95% CI 0.88, 1.09).

**Table 3:** IRRs of AKI during subdivided time periods

Time periods	Adjusted IRRs (95% CI)
Baseline time period	1.00
Total ‘on’ period vs Total ‘off’ period	1.20 (1.15, 1.26)
Initiation period vs Remainder of ‘on’ period	1.26 (1.15, 1.38)
Initiation period vs Baseline ‘off’ quinine	1.48 (1.35, 1.61)
Remainder of ‘on’ period vs Baseline ‘off’ quinine	1.17 (1.11, 1.23)
Pre-exposure period vs Baseline ‘off’ quinine	0.98 (0.88, 1.09)

IRR adjusted for age category.

The initiation period comprised the first 30 days after quinine prescription. The pre-exposure period comprised the 30 days prior to quinine prescription.

## Discussion

To our knowledge, this is the first study to examine the association between quinine exposure and AKI at a population level. We observed a significant association between quinine exposure and AKI in an older population aged 60 years or over using two different study designs applied to the same dataset. In a retrospective longitudinal cohort study examining episodes of care among 12,744 individuals who received community prescriptions for quinine, we found that a prescription for quinine was independently associated with a 27% increased risk of AKI. Similarly, the SCCS demonstrated a 20% increased risk, with quinine initiation being associated with the greatest increased risk of 48%.

Our study has a number of strengths. In our first study design, we examined the association using a large population cohort with median follow-up period of 10 years. By the very nature of its indication, people prescribed quinine for cramps are typically older with comorbidities and other prescribed medications. Access to large national datasets linked by anonymised individual identifiers allowed us to generate a comprehensive profile of an individual’s demographics, comorbidities, community prescriptions and baseline renal function. Adjustment was made based on this information at the time of each episode of care, thereby accounting for the progressive nature of comorbidity in our cohort. Furthermore, the findings of our initial study were supported by the second study design, an SCCS with the results of sensitivity analyses in keeping with the main findings.

However, our study has its limitations inherent to observational studies. Firstly, potentially relevant variables, such as smoking status and medical conditions diagnosed without hospital admission, would not have been captured by our datasets with potential for unmeasured confounding. Secondly, people in our cohort were required to have had creatinine measurements, and testing was inconsistent with some individuals undergoing regular blood tests while others had infrequent and distant serum creatinine measurements. To try and account for this, we adjusted for the residual numbers of episodes of care until the index episode (after adjustment for follow-up time). This adjusted for the number of previous episodes of care (i.e. the number of blood tests) without repeating the adjustment for time of episode since the two were closely correlated. Thirdly, whether or not a medication is taken regularly as prescribed from the date of prescription cannot be determined from observational data. In our first study design, a prescription for quinine within the 12 weeks prior to an episode of care was used to infer recent or current exposure as the majority of quinine prescriptions were either for 28 or 56 tablets. Misclassification of current exposure remains a possibility. Finally, our study is only based within one NHS health board in Scotland, which could affect the generalisability of our results. However, individuals in this region are broadly representative of the national population as a whole, so it is unlikely that our findings would significantly differ.

In the UK, quinine is most commonly prescribed for the treatment of leg cramps but has limited efficacy [8]. Leg cramps are associated with a number of conditions and medications that are also risk factors for AKI, including diuretics, CKD and increased age [8]. For example, leg cramps can occur in the context of volume depletion that may in turn be associated with a prerenal hypoperfusion-related AKI. A degree of overlap is therefore possible and cramps may represent a state of physiological vulnerability. However, quinine itself is also known to affect the kidney through immune-mediated reactions including drug-induced TMA [13]. In one case series of 19 people with confirmed quinine-induced TMA, each person experienced AKI without pre-existing CKD, one individual died and 17 of the remaining 18 required dialysis, three of whom required RRT indefinitely [16]. Although this infrequent acute syndrome is unlikely to confer risk of AKI at a population level, the mechanism of a quinine-dependant antibody reaction could be considered. Other effects of quinine on the cardiovascular system may also play a role. Quinine, the stereoisomer of quinidine, may increase myocardial conduction time and prolong QT interval [21, 22]. It also possesses alpha-adrenergic blocking properties that can predispose to hypotension [23]. In a Danish study of people with heart failure, quinine was associated with an increased risk of death, particularly with concurrent β-blocker therapy [24]. Side effects of quinine can occur at therapeutic doses and include tinnitus, hearing impairment, headache, nausea, vertigo and visual disturbance [8]. Drug interactions can also occur [25]. Older people may be more susceptible to these effects due to an increased elimination half-life associated with age [26] and more prevalent polypharmacy.

Primarily in response to the risk of immune-mediated reactions, regulatory bodies such as the Food and Drug Administration (FDA) and the Medicines and Healthcare products Regulatory Agency (MHRA) have discouraged the prescription of quinine as symptomatic treatment for leg cramps [12, 25]. The MHRA advises that quinine should not be used routinely, its effectiveness should be continually reassessed and trials of treatment discontinuation considered [25]. However, our findings suggest a novel association between community quinine prescriptions and AKI in an older population that is unlikely to be simply related to rare immune-mediated reactions. Whether this relationship is directly related to quinine or the underlying mechanism causing leg cramps remains unclear. Nevertheless, the results of our study support the guidance from the FDA and MHRA. Our findings also highlight to healthcare professionals that patients presenting with cramps should receive careful consideration of the underlying cause, which may be a modifiable risk factor for AKI, prior to initiating symptomatic management. More research is required to characterise further the relationship between quinine, cramps and AKI.

## Supplementary Material

aa-20-0124-File002_afaa079
