# Supplementary material for: Quinine exposure and the risk of acute kidney injury: a population-based observational study of older people
Source: Age Ageing. 2020 May 28;49(6):1042–7. doi: 10.1093/ageing/afaa079 (PMC7583521; doi:10.1093/ageing/afaa079)
Supplement: aa-20-0124-File002_afaa079 [file aa-20-0124-file002_afaa079.docx]

**Quinine exposure and the risk of acute kidney injury: a population based observational study of older people**

**SUPPLEMENTARY DATA**

- Appendix 1: Supplementary Methods
- Appendix 2: Episodes of care and Quinine
- Appendix 3: SCCS Time periods
- Appendix 4: Number of community quinine prescriptions dispensed in Tayside each year
- Appendix 5: AKI Definition
- Appendix 6: ICD-10 codes for SMR01 comorbidities
- Appendix 7: Quinine, AKI, recent prescriptions and comorbidities by episodes of care
- Appendix 8: Baseline characteristics of the SCCS cohort
- Appendix 9: Comorbidities of the SCCS cohort at the start of follow-up

**Appendix 1: Supplementary Methods**

*Data sources*

Anonymised datasets of routinely collected healthcare data were provided by the Health Informatics Centre, University of Dundee. Accurate linkage was enabled by use of the Community Health Index (CHI) number, a unique patient identifier used across Scotland. The following datasets were examined: Serum creatinine values from primary and secondary care were obtained from the laboratory biochemistry system; the General/Acute Inpatient and Day Case - Scottish Morbidity Record (SMR01) was used to identify comorbid conditions from hospital discharges by ICD-10 codes;^1^ prescribed medications dispensed from community pharmacies were extracted from community prescribing data; date of death was ascertained from the Community Health Index dataset (CHI - the NHS Scotland population register), which was also used to identify participant age, sex and postcode-defined socioeconomic status (measured using quintiles of the Scottish Index of Multiple Deprivation);^2^ diabetes status and date of diagnosis were obtained from Scottish Care Information-Diabetes Collaboration (SCI-DC) data;^3^ the Scottish Renal Registry (SRR) was used to identify individuals receiving renal replacement therapy and the date of initiation.^4^

*AKI and CKD Definitions*

AKI and its severity (stages 1 to 3) were defined biochemically based on the Kidney Disease Improving Global Outcomes (KDIGO) criteria^5^ using an algorithm developed by Hapca et al.^6^ AKI was considered present if a creatinine value (termed “index”) fulfilled at least one of three criteria (Appendix 2, Table 1). Creatinine values predating and following this index creatinine measurement were used to define baseline creatinine. The CKD-EPI formula was used to estimate Glomerular Filtration Rate (eGFR) from serum creatinine values and to ascertain a subject’s CKD status.^7^ CKD was defined according to the CKD-KDIGO guideline as eGFR < 60 ml/min per 1.73m^2^ on at least two occasions for more than 90 days.^6,8^ All eGFR values contained within an AKI episode were first removed from the longitudinal data in order to correctly identify CKD status and avoid misclassification between AKI and CKD.

*Co-morbidity*

Co-morbidity at the date of each episode of care was determined from SMR01, community prescribing and biochemistry data. ICD-10 codes from SMR01 data were used to identify a history of myocardial infarction, heart failure, peripheral vascular disease, cerebrovascular disease and liver disease Appendix 2, Table 2).^9^ Concomitant use of other medications, including ACE inhibitors, ARBs, diuretics, NSAIDs, trimethoprim and co-trimoxazole, was defined by the presence of a prescription within the 12 weeks prior to the episode of care. The number of other discrete types of medications dispensed in this time period was also calculated as a measure of polypharmacy. History of previous AKI was identified from serum creatinine measurements from January 2003. This was recorded as the most severe AKI that an individual had previously experienced (i.e. none, AKI stage 1, 2 or 3). CKD status at the date of each episode of care was categorised based on eGFR (eGFR > 60, 30-60, 15-30, < 15 ml/min per 1.73m^2^). The presence or absence of a diagnosis of diabetes (type 1 or type 2) was determined using SCI-DC data.

*Ethics*

Anonymised record linkage was conducted according to the Health Informatics Centre Standard Operating Procedure. The Tayside Research Ethics Committee does not require submission of individual studies that follow this standard operating procedure which is approved by the local Data Protection Officer (Caldicott Guardian).

**References**

1. Information Services Division, NHS National Services Scotland. General Acute Inpatient and Day Case - Scottish Morbidity Record (SMR01). https://www.ndc.scot.nhs.uk/National-Datasets/. Accessed July 8, 2019.

2. Scottish Government. Scottish Index of Multiple Deprivation 2016. https://www.gov.scot/publications/scottish-index-multiple-deprivation-2016/. Accessed July 8, 2019.

3. Scottish Care Information - Diabetes Collaboration (SCI-DC). SCI-Diabetes. https://www.sci-diabetes.scot.nhs.uk/. Accessed July 8, 2019.

4. Scottish Renal Association, NHS National Services Scotland. The Scottish Renal Registry. https://www.srr.scot.nhs.uk/. Accessed July 8, 2019.

5. KDIGO Clinical Practice Guideline for Acute Kidney Injury. *Kidney Int Suppl*. 2012;2(1).

6. Hapca S, Siddiqui M, Kwan R, et al. The relationship between acute kidney injury and chronic kidney disease in a large population with and without diabetes. *Under Review at Kidney Int*. 2019.

7. Levey AS, Stevens LA, Schmid CH, et al. A New Equation to Estimate Glomerular Filtration Rate. *Ann Intern Med*. 2009;150:604-612.

8. KDIGO 2012 Clinical Practice Guideline for Evaluation & Management of CKD. *Kidney Int Suppl*. 2013;3(1).

9. Quan H, Sundararajan V, Halfon P, et al. Coding algorithms for defining comorbidities in ICD-9-CM and ICD-10 administrative data. *Med Care*. 2005;43:1130-1139.

**Appendix 2**

**Figure 1: Episodes of care and Quinine**

**
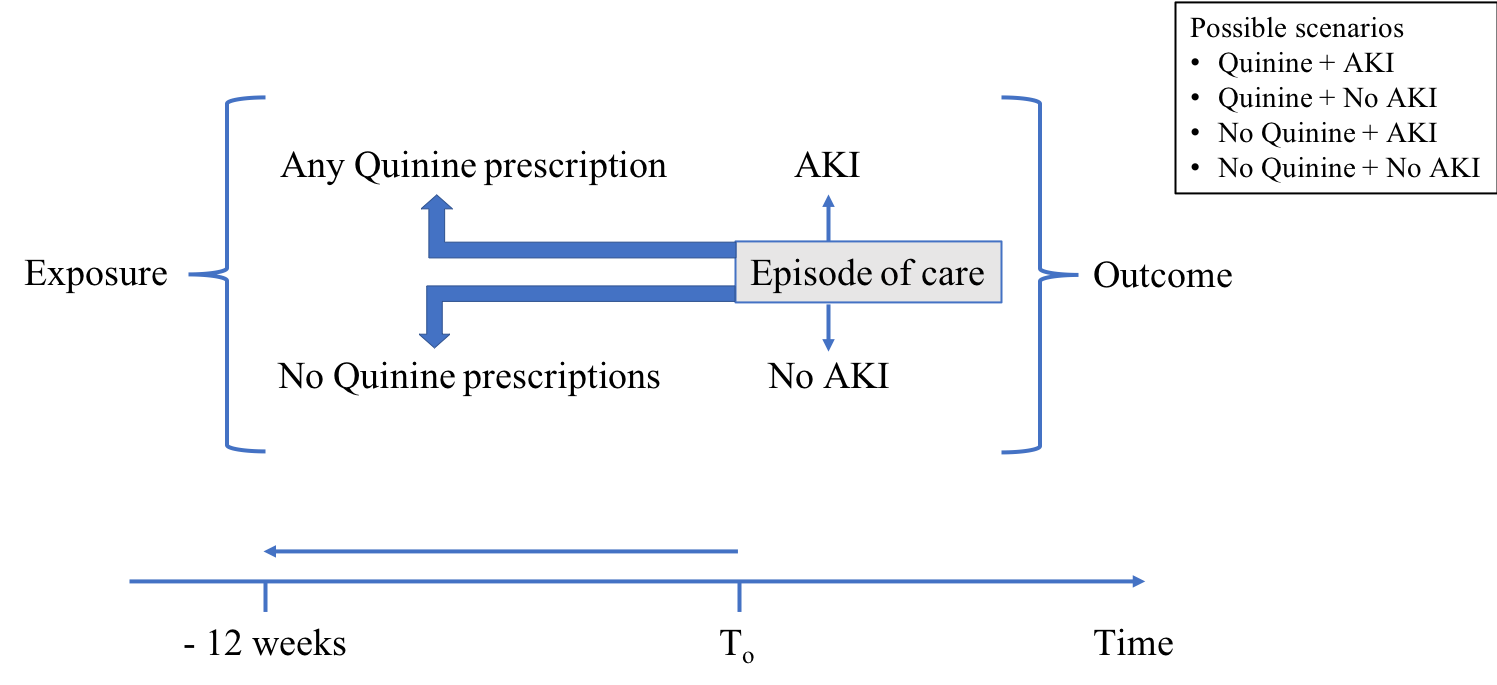
**

*Episode of care: All serum creatinine values from primary or secondary care within 7 days apart. T_o_ is the date of the first creatinine measurement of the episode of care*

**Appendix 3: SCCS Time periods**


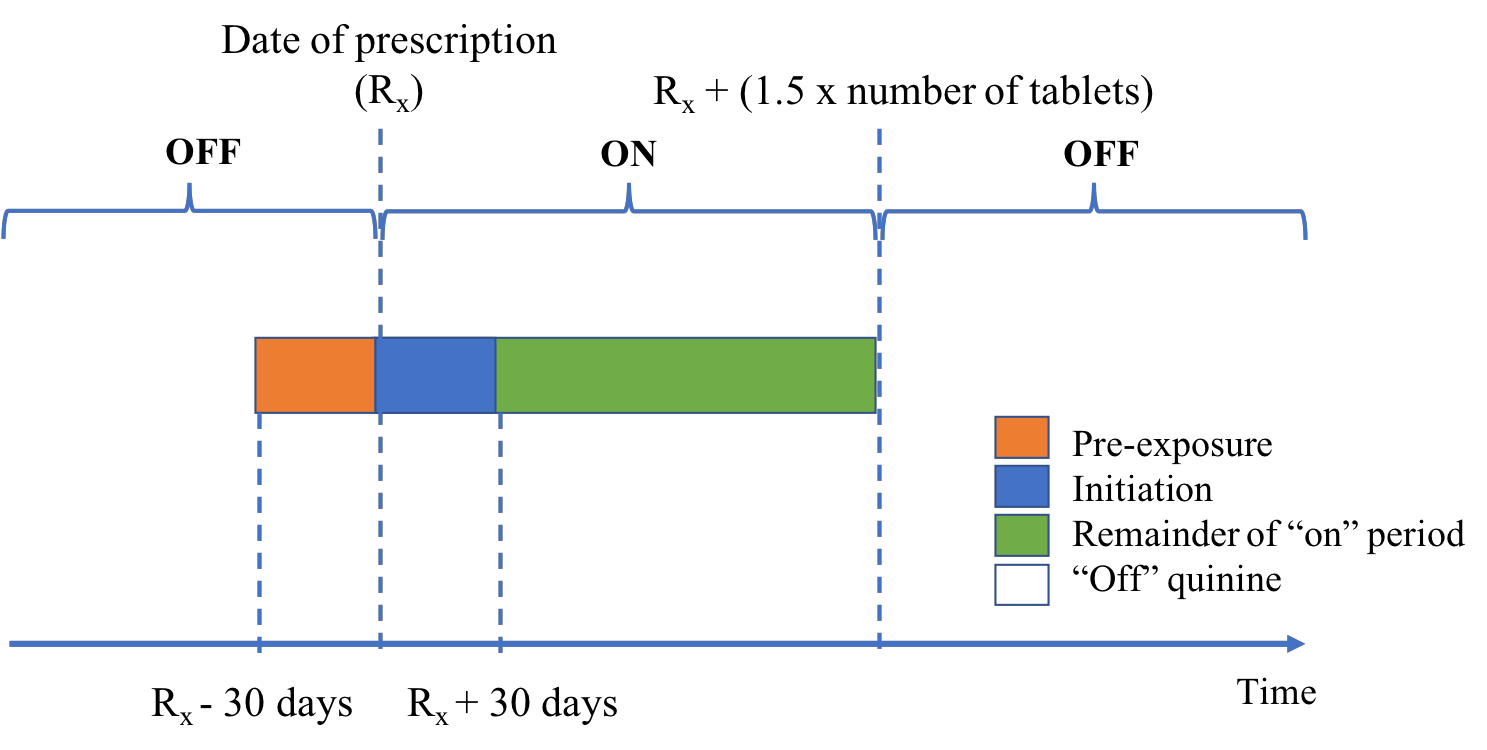


**Appendix 4: Number of community quinine prescriptions dispensed in Tayside each year**

**
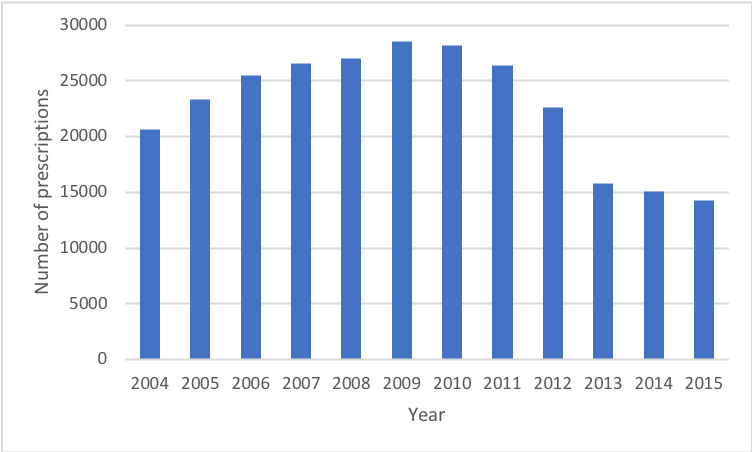
**

**Appendix 5: AKI Definition**

| Criteria | Definition |
| --- | --- |
| 1 | ≥ 26μmol/l greater than lowest creatinine value in the 2 days prior to or post index |
| 2 | ≥ 1.5 times greater than lowest creatinine value in the 7 days prior to or post index |
| 3 | ≥ 1.5 times greater than median of all creatinine values in the 8-365 days prior to or post index |

*A creatinine value (termed “index”) indicated an AKI if it fulfilled any of the above criteria.*

**Appendix 6: ICD-10 codes for SMR01 comorbidities**

| Comorbidity | ICD-10 codes from Quan et al.^32^ | Amendments |
| --- | --- | --- |
| Myocardial Infarction | I21 Acute myocardial infarction  I22 Subsequent myocardial infarction  I25.2 Old myocardial infarction | Added  I23 Current certain complications following acute myocardial infarction |
| Heart Failure | I43 Cardiomyopathy in diseases classified elsewhere (nutritional, infectious…)  I50 Heart failure  I09.9 Rheumatic heart disease, unspecified  I11.0 Hypertensive heart disease with (congestive) heart failure  I13.0 Hypertensive heart and renal disease with (congestive) heart failure  I13.2 Hypertensive heart and renal disease with (congestive) both heart and renal failure  I25.5Ischaemic cardiomyopathy  I42.0 Dilated cardiomyopathy  I42.5 Other restrictive cardiomyopathy  I42.6 Alcoholic cardiomyopathy  I42.7 Cardiomyopathy due to drugs and other external agents  I42.8 Other cardiomyopathy  I42.9 Cardiomyopathy unspecified  P29.0 Neonatal cardiac failure |  |
| Peripheral Vascular Disease | I70 Atherosclerosis  I71 Aortic aneurysm and dissection  173.1 Thromboangiitis obliterans (Buerger)  173.8 Other specified vascular disease  173.9 Peripheral vascular disease unspecified  I77.1 Stricture of artery  K55.1 Chronic vascular disorders of intestines  K55.8 Other vascular disorders of intestines  Z95.8-9 Presence of other cardiac or vascular graft | Added  I72 Other aneurysm and dissection  I74 Arterial embolism and thrombosis  K55.0 Acute vascular disorders of intestines |
| Cerebrovascular Disease | G45 Transient ischaemic attacks and related syndromes  G46 Vascular syndromes of brain in cerebrovascular disease  I60-9 Cerebrovascular disease  H34 Retinal vascular occlusions |  |
| Liver Disease | B18 Chronic viral hepatitis  K70 Alcoholic liver disease  K71 Toxic liver disease (excluding K71.2 with acute hepatitis and K71.6, K71.8, K71.9)  K72.1 Chronic hepatic failure  K72.9 Hepatic failure unspecified  K73 Chronic hepatitis not elsewhere classified  K74 Fibrosis and cirrhosis of liver  K76 Other diseases of liver (excluding 76.1)  Z94.4 Liver transplant status | Added  K71.6 Toxic liver disease with hepatitis not elsewhere specified  K71.8 Toxic liver disease with other disorders of liver  K71.9 Toxic liver disease, unspecified  K76.1 Chronic passive congestion of liver |

**Appendix 7: Quinine, AKI, recent prescriptions and comorbidities by episodes of care**

|  |  | All episodes  N=267,900 | Episodes with AKI  N=13,616 | Episodes with AKI and Quinine  N=5,096 |
| --- | --- | --- | --- | --- |
| Variable |  | **Frequency (%)** | **Frequency (%)** | **Frequency (%)** |
|  | Quinine | 79,234 (29.6) | 5,096 (37.4) | 5,096 (100) |
|  |  |  |  |  |
| AKI | AKI | 13,616 (5.1) | 13,616 (100) | 5,096 (100) |
|  | Stage 1 | 10,279 (75.5) | 10,279 (75.5) | 3,865 (75.8) |
|  | Stage 2 | 2,338 (17.2) | 2,338 (17.2) | 824 (16.2) |
|  | Stage 3 | 999 (7.3) | 999 (7.3) | 407 (8.0) |
|  |  |  |  |  |
| Age | Mean (SD) | 76.0 (8.2) | 78.8 (8.4) | 78.8 (8.2) |
|  |  |  |  |  |
| Other prescriptions | ACEI | 83,744 (31.3) | 4,368 (32.1) | 1,775 (34.8) |
|  | ARB | 39,115 (14.6) | 1,856 (13.6) | 638 (12.5) |
|  | Diuretic | 125,038 (46.7) | 7,978 (58.6) | 3,255 (63.9) |
|  | NSAID | 30,536 (11.4) | 1,489 (10.9) | 678 (13.3) |
|  | Trimethoprim | 17,801 (6.6) | 1,807 (13.3) | 582 (11.4) |
|  | Mean no. of other drugs (SD) | 4.1 (2.5) | 4.8 (2.6) | 5.2 (2.5) |
|  |  |  |  |  |
| History of previous AKI | Nil | 181,108 (67.6) | 5,471 (40.2) | 2,007 (39.4) |
|  | Stage 1 | 58,955 (22.0) | 4,846 (35.6) | 1,863 (36.6) |
|  | Stage 2 | 18,710 (7.0) | 2,135 (15.7) | 786 (15.4) |
|  | Stage 3 | 9,127 (3.4) | 1,164 (8.6) | 440 (8.6) |
|  |  |  |  |  |
| CKD status | eGFR >60 | 140,827 (52.6) | 4,787 (35.2) | 1,593 (31.3) |
|  | eGFR 30-60 | 105,897 (39.5) | 6,731 (49.4) | 2,604 (51.1) |
|  | eGFR 15-30 | 19,024 (7.1) | 1,750 (12.9) | 720 (14.1) |
|  | eGFR <15 | 2,152 (0.8) | 348 (2.6) | 179 (3.5) |
|  |  |  |  |  |
| Comorbidities | Type 1 Diabetes Mellitus | 860 (0.3) | 59 (0.4) | 21 (0.4) |
|  | Type 2 Diabetes Mellitus | 66,358 (24.8) | 3,537 (26.0) | 1,395 (27.4) |
|  | Myocardial Infarction | 31,764 (11.9) | 2,612 (19.2) | 996 (19.5) |
|  | Heart Failure | 25,792 (9.6) | 2,836 (20.8) | 1,022 (20.1) |
|  | Peripheral Vascular Disease | 22,231 (8.3) | 2,150 (15.8) | 899 (17.6) |
|  | Cerebrovascular Disease | 29,530 (11.0) | 2,628 (19.3) | 1,007 (19.8) |
|  | Liver Disease | 5,296 (2.0) | 480 (3.5) | 170 (3.3) |

*Abbreviations: AKI Acute Kidney Injury, SD Standard deviation, ACEI Angiotensin Converting Enzyme Inhibitor, ARB Angiotensin II Receptor Blocker, NSAID Non-Steroidal Anti-Inflammatory Drug, CKD Chronic Kidney Disease, eGFR estimated Glomerular Filtration Rate (ml/min per 1.73m^2^). Trimethoprim refers to antibiotics containing trimethoprim including co-trimoxazole. Age at episode of care.*

**Appendix 8: Baseline characteristics of the SCCS cohort**

| Variable |  | Frequency (%)  N=5,907 |
| --- | --- | --- |
| Sex | Female | 3,695 (62.6) |
|  | Male | 2,212 (37.4) |
|  |  |  |
| Age | Median (IQR) | 73.6 (67.1-79.8) |
|  |  |  |
| SIMD | 1 | 1,128 (19.4) |
|  | 2 | 1,003 (17.2) |
|  | 3 | 1,078 (18.5) |
|  | 4 | 1,706 (29.3) |
|  | 5 | 903 (15.5) |
|  | Missing data | 89 |
|  |  |  |
| Follow-up | Median in years (IQR) | 8.9 (5.4-11.3) |
|  | RRT during study period | 35 (0.6) |
|  | Died during study period | 3,642 (61.7) |
|  |  |  |
| Quinine prescriptions | Total | 121,518 |
|  | Median per person (IQR) | 8 (2-32) |
|  | Median no. of tablets per prescription (IQR) | 30 (28-56) |
|  |  |  |
| AKI | Total | 13,616 |
|  | Median per person (IQR) | 2 (1-3) |
|  | Stage 1 | 10,279 (75.5) |
|  | Stage 2 | 2,338 (17.2) |
|  | Stage 3 | 999 (7.3) |

*Abbreviations: IQR Interquartile Range (presented as Q1-Q3), SIMD Scottish Index of Multiple Deprivation (Quintiles 1-most deprived, 5-least deprived), RRT Renal Replacement Therapy. Age at start of follow-up.*

**Appendix 9: Comorbidities of the SCCS cohort at the start of follow-up**

| Variable |  | Frequency (%)  N=5,907 |
| --- | --- | --- |
| CKD status | eGFR >60 | 4,920 (83.3) |
|  | eGFR 30-60 | 880 (14.9) |
|  | eGFR 15-30 | 102 (1.7) |
|  | eGFR <15 | 5 (0.1) |
|  |  |  |
| History of previous AKI | Nil | 5,417 (92.6) |
|  | Stage 1 | 317 (5.4) |
|  | Stage 2 | 85 (1.4) |
|  | Stage 3 | 34 (0.6) |
|  |  |  |
| Comorbidities | Type 1 Diabetes Mellitus | 16 (0.3) |
|  | Type 2 Diabetes Mellitus | 915 (15.5) |
|  | Myocardial Infarction | 388 (6.6) |
|  | Heart Failure | 298 (5.0) |
|  | Peripheral Vascular Disease | 306 (5.2) |
|  | Cerebrovascular Disease | 381 (6.5) |
|  | Liver Disease | 63 (1.1) |

*Abbreviations: CKD Chronic Kidney Disease, eGFR estimated Glomerular Filtration Rate (ml/min per 1.73m^2^), AKI Acute Kidney Injury*
